# Aluminum and aluminum oxide nanomaterials uptake after oral exposure - a comparative study

**DOI:** 10.1038/s41598-020-59710-z

**Published:** 2020-02-14

**Authors:** Benjamin C. Krause, Fabian L. Kriegel, Daniel Rosenkranz, Nadine Dreiack, Jutta Tentschert, Harald Jungnickel, Pegah Jalili, Valerie Fessard, Peter Laux, Andreas Luch

**Affiliations:** 1 German Federal Institute for Risk Assessment (BfR), Department of Chemical and Product Safety, Max-Dohrn-Straße 8-10, 10589 Berlin, Germany; 2 ANSES, French Agency for Food, Environmental and Occupational Health and Safety, Fougères Laboratory, 10B rue Claude Bourgelat, 35306 Fougères Cedex, France

**Keywords:** Metals, Nanoparticles, Analytical chemistry

## Abstract

The knowledge about a potential *in vivo* uptake and subsequent toxicological effects of aluminum (Al), especially in the nanoparticulate form, is still limited. This paper focuses on a three day oral gavage study with three different Al species in Sprague Dawley rats. The Al amount was investigated in major organs in order to determine the oral bioavailability and distribution. Al-containing nanoparticles (NMs composed of Al^0^ and aluminum oxide (Al_2_O_3_)) were administered at three different concentrations and soluble aluminum chloride (AlCl_3_·6H_2_O) was used as a reference control at one concentration. A microwave assisted acid digestion approach followed by inductively coupled plasma mass spectrometry (ICP-MS) analysis was developed to analyse the Al burden of individual organs. Special attention was paid on how the sample matrix affected the calibration procedure. After 3 days exposure, AlCl_3_·6H_2_O treated animals showed high Al levels in liver and intestine, while upon treatment with Al^0^ NMs significant amounts of Al were detected only in the latter. In contrast, following Al_2_O_3_ NMs treatment, Al was detected in all investigated organs with particular high concentrations in the spleen. A rapid absorption and systemic distribution of all three Al forms tested were found after 3-day oral exposure. The identified differences between Al^0^ and Al_2_O_3_ NMs point out that both, particle shape and surface composition could be key factors for Al biodistribution and accumulation.

## Introduction

Aluminum is one of the most abundant elements on earth and is widely used in many different consumer product applications due to its unique characteristics. As an ubiquitous element, Al occurs in natural sources, e.g. food and drinking water, as well as in food additives, packaging and kitchenware^1,2^. Besides industrial, also agricultural, medical and consumer product uses are known for synthetic alumina, mixed Al silicate and Al oxide NMs. Especially composite materials containing Al show favourable properties when being used as packaging materials for the protection of food against humidity and oxidation and are more and more common^3–6^. Moreover, various Al salts are used in food as additives, e.g. as stabilizers, pH regulators and anti-caking agents^7,8^. As a consequence of modern life style Al-containing materials and substances are of high abundance in the human environment. In our study we could show *de novo* synthesis of Al-containing nanomaterials from ions after induction of a pH shift^9^. Based on this and because of the detection and quantification of Al in biological media is difficult, one should assume at least partial particle concentration in the body after oral uptake of Al. This raises questions about the potential hazard of particulate Al species.

Adverse effects of Al have been repeatedly discussed in the past. Neurodegenerative diseases such as Alzheimer’s^10,11^ as well as certain bone diseases^12–14^ and dialysis dementia^15,16^ have been attributed to Al exposure. Although inhalation and dermal uptake of Al may occur, ingestion is mainly responsible for Al organ burden with regard to the public population^17^. Consequently, a tolerable weekly intake (TWI) of 1 mg/kg body weight was implemented by the European Food Safety Authority (EFSA), while a provisional tolerable weekly intake (PTWI) of 2 mg/kg body weight has been set by the Joint FAO/WHO Expert Committee on Food Additives (JECFA). However, both values do not distinguish between ionic and nanoparticulate Al species^18,19^, despite the increasing use of Al-containing NMs^20^. Al NMs may change the impact on human health due to their unique physico-chemical properties, e.g. their size, high specific surface area, and high reactivity^21–24^. Hence, reliable information on oral uptake and the resulting organ burden of Al-containing NMs is needed. In a recent *in vitro* study, the influence of an artificial digestion process on two different Al NMs and one salt species was examined^9^. This process led to the *de novo* formation of nanoparticles in the artificial intestinal juice for the two NM species and also for the Al salt^9^. *In vivo* studies in rats showed that ionic Al is systemically distributed throughout the organs after oral uptake. High concentrations were found in kidneys, liver, spleen, bones and brain. Al is also highly persistent with half-life times up to 150 days, for example, in the brain^25–28^. On the contrary, little is known about the distribution of particulate Al species, and possible differences between individual Al species.

In this study, we compared Al^0^ and Al_2_O_3_ NMs with an ionic Al species. Oral uptake was investigated by measuring Al concentrations in duodenum and colon samples. Systemic exposure was estimated through analyses of blood, liver, kidney and spleen samples. We carefully examined matrix suppression effects since they may affect the Al levels detected by ICP-MS^29^. For this purpose, a calibration for each organ was compared with the standard aqueous calibration method. Our approach highlights the advantages of matrix-matching in ICP-MS based *in vivo* studies. A reduction of the number of animals needed for a robust analysis combined with low limits of detection were achieved, while at the same time, matrix effects were taken into account.

## Results

### Characterization of Al and Al_2_O_3_ NMs

Both, Al^0^ and Al_2_O_3_ NMs have been characterized elsewhere (Table 1)^30^. In brief, the core particle diameter was confirmed to be between 2–50 nm for the nearly spherical Al^0^ NMs by transmission electron microscopy (TEM). Al_2_O_3_ NMs are more rod-shaped with a width mostly around 10 nm and a length that varies between 20–50 nm as determined by TEM. The size distribution showed a broader variance for Al^0^ than for Al_2_O_3_ NMs. Dynamic light scattering (DLS) determined the hydrodynamic diameter in dispersion of 0.05% BSA. For Al^0^ NMs, a hydrodynamic diameter of 250 nm was detected, while Al_2_O_3_ NMs showed a smaller diameter of 180 nm. X-ray diffraction (XRD) and TEM in an element resolved mode demonstrated the difference of the Al^0^ and Al_2_O_3_ NMs composition and especially their surface. While Al^0^ NMs had a core-shell structure with Al core and a 2–5 nm oxygen shell, the Al_2_O_3_ NMs were homogeneously oxidized. An overview about the main characterization data is given in Table 1. An artificial digestion approach showed a decreased solubility coupled to *de novo* formation of Al-containing particles in the nanoscale range occurring in the intestinal fluid compared to the stomach^9^.

**Table 1:** Characterization data for Al^0^ and Al_2_O_3_ NM. Modified from^30^.

Methods	Al^0^ NM	Al_2_O_3_ NM
TEM	Primary particle size and shape: 2–50 nm, nearly spherical	Primary particle size and shape: 10 × 20–50 nm, grain-like shape
EELS-TEM	Core-shell structure, thin (2–5 nm) oxide layer	Fully oxidized particle
XRD	Aluminum surface; partially oxidized	Fully oxidized surface
SAXS	Particle radius: >10 nm	Primary particle radius: 7.1 nm Aggregates’ radius: >10 nm
SP-ICP-MS	Primary particle size: 54–80 nm	Primary particle size: 50–80 nm
ICP-MS	Ion release: 0.2–0.5% (in 0.05% BSA)	Ion release: 0.2–0.4% (in 0.05% BSA)
ToF-SIMS	Particle-amino acid agglomerates	Particle-amino acid agglomerates; polyoxo-aluminates
IBM, atom number %	Impurities: P (1%); biocorona: S (5%), protein; Ca_3_(PO_4_)_2_ coating: P (3%)	Impurities: S (0.2%); biocorona: S (1%), Ca_3_(PO_4_)_2_ coating: P (1%)

### Matrix-matching

After an in-house validation of the analytical method for digestion, quantification and evaluation of the Al content in the investigated organs, an ANOVA and PCA analysis based on the different treatment groups were performed (see Methods section for details). This step was conducted to exclude the possibility that animals did not correspond to the variation in the overall population. The results of ANOVA and PCA (see Supplementary Table S1 and Supplementary Fig. S1) showed no significant difference among the animals of the different treatment groups.

**Figure 1:**
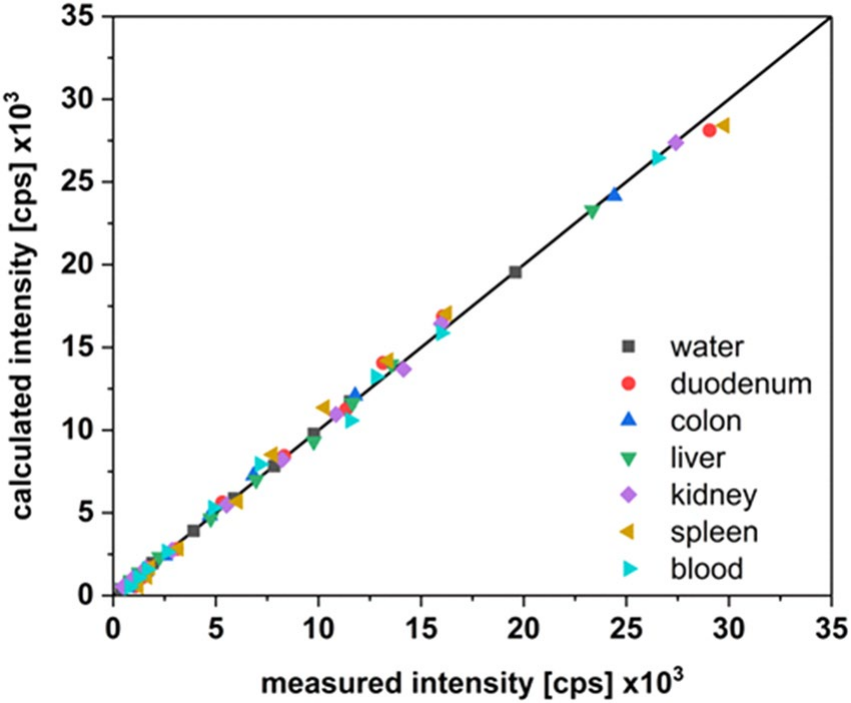
Regression analysis of matrix calibration data. The measured intensities were plotted versus the calculated intensities which were obtained from the regression functions.

In order to minimize measurement uncertainties and standard deviation for the quantification, a matrix calibration was carried out for each organ investigated. The calibration points quantified in the different matrices were highly correlated to the data points calculated from the regression curve of each organ (Fig. 1 and Table 2). For each tested organ a calibration linearity between 0.9960 and 0.9997 was achieved.

**Table 2:** Analytical figure of merit. The different matrices (water, duodenum, colon, liver, kidney, spleen and blood) were evaluated based on the quality parameters of the calibration (linearity, sensitivity, limit of detection (LOD), limit of quantification (LOQ), recovery). F-Test was carried out to compare the variance between water and matrix calibration sensitivities.

Matrix	Linearity (r²)	Sensitivity [cps (ng g^−1^)^−1^]	LOD [µg g^−1^] organ	LOQ [µg g^−1^] organ	F-Test*	Recovery [%]
water	0.9994	390.68	0.035	0.042	1	100
duodenum	0.9979	526.35	0.070	0.115	0.13	135
colon	0.9995	483.10	0.466	1.394	0.56	92
liver	0.9996	465.66	0.024	0.049	0.44	96
kidney	0.9997	568.27	0.032	0.082	0.16	122
spleen	0.9959	547.35	0.039	0.082	0.13	96
blood	0.9983	528.93	0.008	0.015	0.20	97

*F-Test: water/matrix; F ≥ 0.99 for using water calibration only.

**Figure 2:**
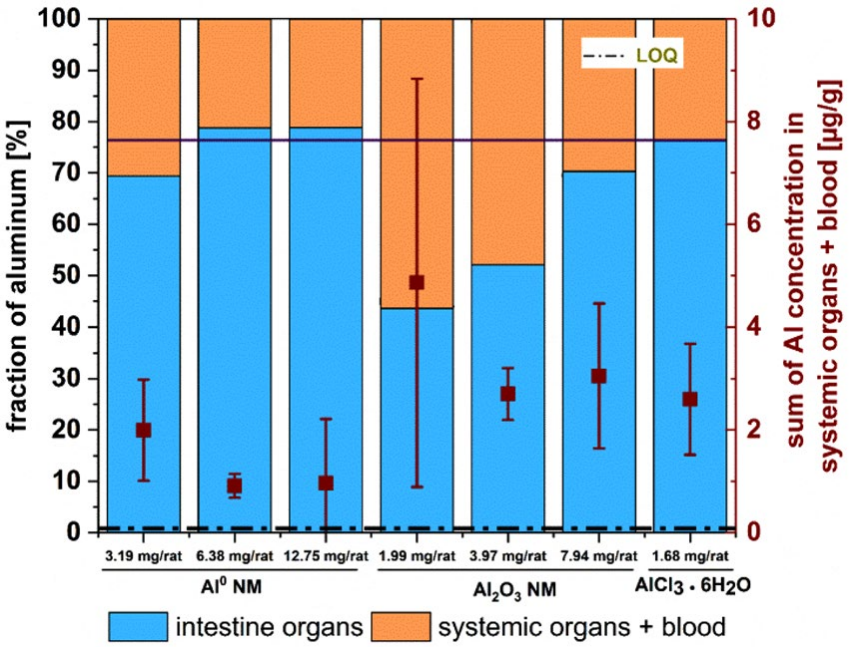
Overall fraction of Al in investigated organs. The intestine (duodenum and colon; blue) and the systemic organs (liver, kidney, spleen) and blood (orange) values [%] are given. The black bar indicates the reference value found for ionic AlCl_3_·6H_2_O fraction [%]. Brown squares indicate the median of the sum of Al concentration in systemic organs and blood [µg/g] and the vertical bars indicate the interquartile ranges.

The F-Test compares the variances of both water and matrix calibration sensitivities. This revealed significant differences between the calibration in water and in the several matrices. It is therefore not possible to use the water calibration without correction factors calculated from the matrix calibration. In order to avoid the preparation of matrix calibration for each day of measurement a daily response factor (DRF) was used. The DRF accounts for day to day ICP-MS sensitivity variations as well as for matrix effects. After the introduction of the DRF, all sensitivities of the matrix calibrations could be correlated to the water calibration of each day to calculate the recovery rate. The intensities for Al in water are considerably higher than those of each matrix used. Thus, the values for recovery depend on the matrix (see Supplementary Fig. S2). These results pointed out the necessity for a matrix-matched calibration, which led to reliable results. The sample intensities were corrected by subtracting the value of averaged microwave blanks (see Eq. (1) in the methods section). In order to avoid performing a matrix calibration curve each day, a matrix calibration was done on one day and the resulting intensities of the samples were corrected using a DRF (see Eq. (2) in the methods section). We defined the sensitivity of the ICP-MS device as the slope of the calibration curve (Table 2). Therefore, the sensitivity obtained using a water calibration on the day when the samples were analysed was divided by the sensitivity of the water calibration from the day of the matrix calibration (see Eq. (3) in the methods section). Additionally, the recovery of the microwave-assisted digestion was calculated. Considering a factor taking into account these corrections, it was possible to calculate the concentration of Al burden per gram of tissue in a highly robust manner (see Eqs. (4–6) in the methods section). All obtained values were above the LOQ (Fig. 2). Assuming that the data are not equally distributed, through median and average comparison (Table 3), the Wilcoxon sign test was used for statistical analysis of non-normal distributed dependent groups. For each dose group, the distribution of Al concentrations in the organs was examined. The significance levels were corrected according to Holm-Šidàk and an unspecific pairwise comparison was carried out^31^. For almost all comparisons, a significant difference between dose groups and the Al species as well as to the control group at the significance level of 0.05 (one-side) could be observed.

**Table 3:** Comparison of median and mean for determination of skewness for doses per kg body weight. “All organs” refers to all examined organs.

Dose	∑ all organs			∑ Liver, Kidney, Spleen		
	Median µg/g	Mean µg/g	Skewness	Median µg/g	Mean µg/g	Skewness
Al^0^ 6.25 mg/kg	8.16	10.21	right skew	2.46	3.29	right skew
Al^0^ 12.5 mg/kg	3.85	4.88	right skew	0.72	1.30	right skew
Al^0^ 25 mg/kg	5.79	9.56	right skew	1.22	2.54	right skew
Al_2_O_3_ 6.25 mg/kg	11.16	25.83	right skew	5.01	6.78	right skew
Al_2_O_3_ 12.5 mg/kg	10.08	16.09	right skew	4.81	5.84	right skew
Al_2_O_3_ 25 mg/kg	15.37	19.80	right skew	4.58	5.84	right skew
AlCl_3_·6H_2_O 25 mg/kg	9.38	14.23	right skew	2.33	6.85	right skew

### Uptake evaluation

In this study, AlCl_3_·6H_2_O is regarded as a reference substance to determine the uptake of Al ions. According to the mass balance in all investigated organs, ca. 25% of the ionic AlCl_3_·6H_2_O did pass the intestinal barrier (Fig. 2). A similar behaviour was observed for the Al^0^ NMs at all three concentrations tested although the total Al content administered with Al^0^ NMs was 1.9–7.6-fold higher when compared to AlCl_3_·6H_2_O. If the overall uptake of Al was comparable for the Al^0^ NM and the salt species, in contrast, a higher overall Al uptake could be observed in animal tissues treated with Al_2_O_3_ NMs (Fig. 2). It is worth noting that the accumulation of Al in the intestine (washed duodenum and colon) and in systemic organs differed significantly. In contrast to Al^0^ treatment, a concentration-dependent increase of intestinal Al was detected after Al_2_O_3_ administration.

Indeed, in the intestine, a dose-dependent linear increase of the Al fraction was recorded. Figure 2 depicts the fractions of Al found in intestine and systemic organs investigated in this study. The sum of both Al fractions was normalized to 100%.

**Figure 3:**
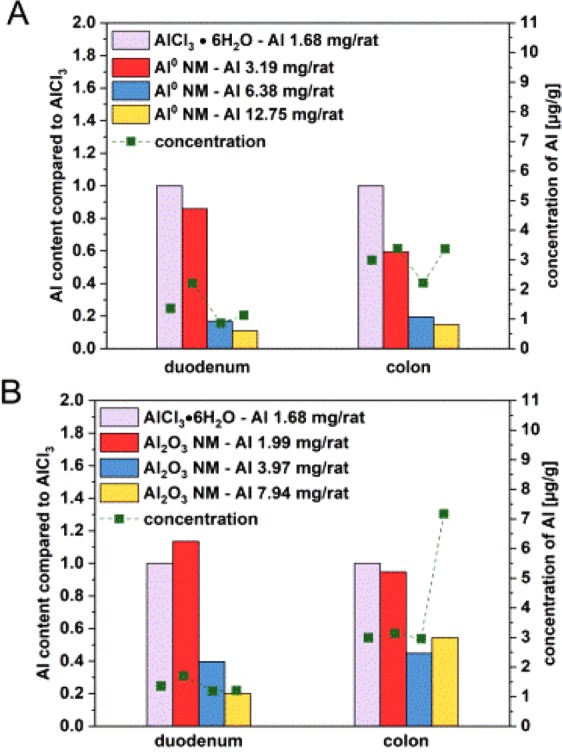
Al concentrations in duodenum and colon after 3 day oral treatment. The organs were collected 3 hours after the last administration of Al^0^ NMs (**A**) and Al_2_O_3_ NMs (**B**). Each bar was normalized on the concentration obtained with AlCl_3_·6H_2_O at 1.7 mg/rat corrected by the corresponding Al content of AlCl_3_·6H_2_O and the applied NM. Green squares display the Al concentration which was found in the organ [µg/g].

After administration of Al^0^ NMs, the lowest dose of 3.19 mg/rat resulted in the highest concentrations in intestine, both duodenum and colon with concentrations in the range of 1–2 µg/g and of 2–3.5 µg/g respectively (Fig. 3A).

The Al uptake was calculated based on the mass fraction of Al in the compound. The Al concentration was then median normalized to the values found in samples with a 1.68 mg/rat AlCl_3_·6H_2_O exposure (see Eq. (7) in the methods section, for further details see Supplementary Tables S2 and S3). An overview about all investigated organs with the Al concentration in µg/g for all eight treatment groups is given in Supplementary Fig. S3. The application of Al^0^ NM at the lowest dose resulted in the highest Al concentration in the duodenum. Normalized to the Al concentration found in the same organs following exposure to AlCl_3_·6H_2_O, only 85% were detected. A less prominent concentration was determined relatively to the AlCl_3_·6H_2_O for the mid (about 17%) and the high dose (about 11%). A comparable effect was observed in the colon, where the relative amount was about 60% for the lowest dose, 20% for the mid and about 15% for the highest dose (Fig. 3A). Comparing Al^0^ NMs and Al_2_O_3_ NMs, more Al could be detected in the duodenum after Al_2_O_3_ exposure. However, Al_2_O_3_ NMs contain only about 62% of the Al content than Al^0^ NMs. While no dose dependency was observed in the duodenum, the Al concentration increased dose dependently in the colon. A maximum concentration of 7.7 µg/g organ was observed at the highest dose level (Fig. 3B). Normalized to an ionic AlCl_3_·6H_2_O dose of 1.68 mg Al/rat, the treatment with Al_2_O_3_ NMs resulted in an Al content of 20–113% in the duodenum and of 45–95% in the colon (Fig. 3B).

**Figure 4:**
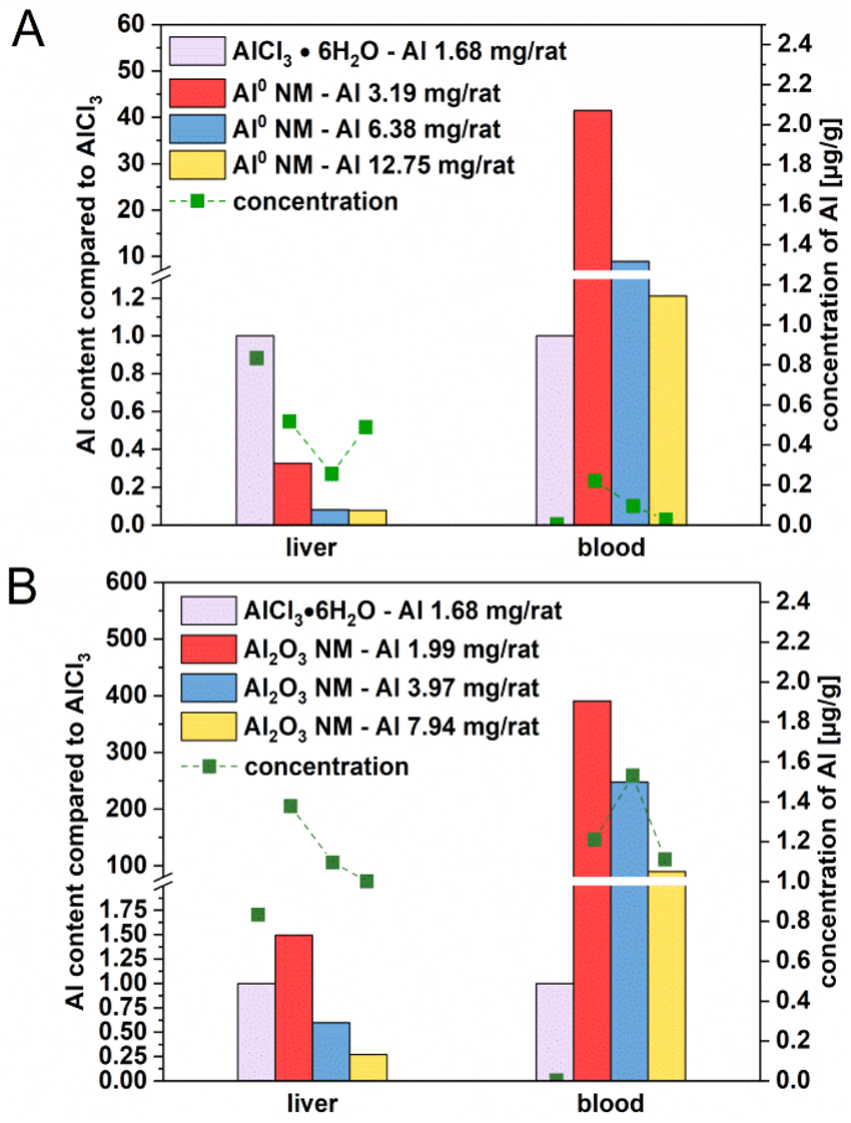
Accumulation of Al in liver and blood after 3 day oral treatment. All organs were collected 3 hours after the last administration of Al^0^ NMs (**A**) and Al_2_O_3_ NMs (**B**). Each bar was normalized on the concentration of 1.7 mg Al/rat. Values were corrected by the corresponding Al content of AlCl_3_·6H_2_O and the applied NM. Green squares display the Al concentration which was found in the organ in [µg/g].

After crossing the intestinal barrier, the different Al species were distributed to systemic organs via blood. At the lowest dose of Al^0^ NM, Al was detected to a large extent in liver and blood (Fig. 4A), and in the two intestinal sections (Fig. 3A). Compared to AlCl_3_·6H_2_O, a lower amount of Al was detected in liver (0.07–0.33-fold of the amount detected for AlCl_3_·6H_2_O), while a 8.98–41.45-fold increased uptake was measured in blood for mid and low doses of Al^0^ NMs (Fig. 4A).

A similar concentration-dependent behaviour (in respect to the amount given) was observed for Al_2_O_3_ NMs: the lowest dose showed the highest difference in comparison to the median normalized and fraction-corrected concentration of AlCl_3_·6H_2_O. However, the concentration dependency showed a linear decrease (Fig. 4B). Compared to the corresponding values in AlCl_3_·6H_2_O treated animals, the Al content varied between 1.49-fold for the lowest and 0.27-fold for the highest dose (liver). In blood, an almost 390.92-fold increase for the lowest dose and a 89.92-fold increase for the highest dose were observed in comparison to animals treated with AlCl_3_·6H_2_O (Fig. 4B). Al concentrations in liver and blood were found between 1.07 and 1.64 µg/g for Al_2_O_3_ NMs treated rats, compared to a range between 0.03 and 0.52 µg/g for Al^0^ NM treated rats. When comparing Al^0^ and Al_2_O_3_ NMs, the latter one is found up to ten times higher in the blood (Fig. 4).

**Figure 5:**
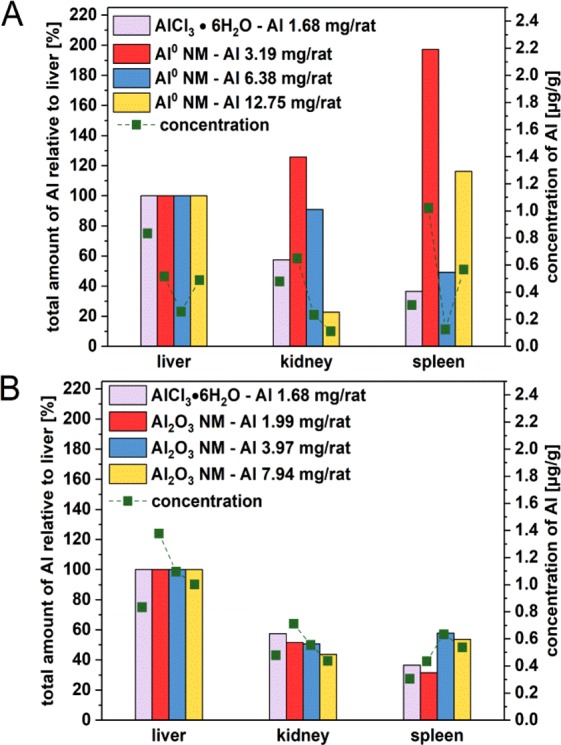
Comparison of the Al content of liver, kidney and spleen after 3 day oral treatment. The organs were collected 3 hours after the last administration of Al^0^ NMs (**A**) and Al_2_O_3_ NMs (**B**). Each bar was normalized on the uptake of the liver in the corresponding treatment group. Green squares display the Al concentration which was found in the respective organ in [µg/g].

Since liver is the first organ after intestinal uptake, a significant accumulation of Al was expected. Therefore, we normalized the liver uptake as 100% to show the different uptake and accumulation patterns of Al^0^ and Al_2_O_3_ NMs at various concentrations. With the low and medium Al^0^ NM doses, a relatively high concentration of Al was found in the kidney with concentrations of 0.65 and 0.23 µg/g respectively. However, with Al^0^ NMs, the maximum Al concentration (1.02 µg/g) was found in the spleen with the lowest treatment dose (Fig. 5A). Al_2_O_3_ NMs accumulated three times higher in the liver than Al^0^ NMs. Following to administration of Al_2_O_3_ NMs, the Al levels detected in the liver (1.07 to 1.48 µg/g) are the highest among the other systemic organs (Fig. 5B).

## Discussion

In the current study we compared the oral bioavailability and distribution of ionic and nanoparticulate Al^0^ and Al_2_O_3_ in rats following a 3-day oral treatment. ICP-MS was used to quantify the Al concentration in duodenum, colon, blood, liver, kidney and spleen to evaluate the biodistribution patterns of the three Al species. Some challenges were faced for the quantification of Al amounts. First, the ubiquitous presence of Al in the tissues hinders the detection of Al that originates from the treatment. The background levels of Al were shown to vary from organ to organ (see Supplementary Fig. S3). For this purpose, a robust calibration methodology was developed where matrix effects were taken into account. Our matrix calibration data clearly indicated the need for matrix correction (see Supplementary Fig. S2). In fact, the calibration in water showed a higher sensitivity compared to all calibrations in matrices. The matrix affects the analyte sensitivity due to signal suppression or enhancement by dissolved salts. Another possible interference especially in high matrix load samples is the deposition of solid matrix on the ICP-MS apertures^32^. After setting up the matrix calibration method, we were able to detect very low concentrations of Al in the tissue samples above the background levels (see Table 2 and Supplementary Fig. S3). However, as daily matrix calibration requires an analytical effort and the recurrent use of animal tissues, the DRF, an established approach in chromatography, was adapted to this study. This approach not only compensates for daily measurement fluctuations but also adjusts the sensitivity of the calibration in water by the calibration with matrix in order to take matrix effects into account. The DRF methodology contributes to achieve the high sensitivity required for a reliable detection of low Al levels by ICP-MS. Consequently, we recommend always testing the influence of sample matrix on the analyte detection. If, as demonstrated in this study, analyte suppression is observed, matrix calibration in combination with the DRF approach is advised for sample analysis.

Following oral administration by gavage with Al^0^ NM, Al_2_O_3_ NM and ionic aluminum, Al levels were detected in all investigated organs and specific differences were highlighted between Al^0^ and Al_2_O_3_ NMs compared to ionic AlCl_3_·6H_2_O. These findings are in contrast to a previous inhalation study where, despite high lung burdens of AlOOH NMs, no significant systemic organ burden (brain, liver, and kidney) could be detected^33^. This highlights the importance of the oral uptake route for the overall Al burden in humans.

After gavage the distribution of Al_2_O_3_ NM between intestine and systemic organs showed significant differences (Fig. 2), while distribution of Al^0^ NM was rather comparable to the ionic AlCl_3_·6H_2_O. Therefore, NMs with the same metallic component may show different accumulation behaviour in living organisms. We observed a dose-dependent decrease of Al levels in systemic organs with increasing concentrations of Al_2_O_3_ NM suggesting an impairment of the intestinal barrier function with increasing dose and subsequent accumulation in the intestine. An explanation could be that the agglomeration state of Al_2_O_3_ NMs was higher with increasing concentrations. Above a certain size, agglomerates may be less prone to cross the gut-blood barrier. Therefore not only the composition of the NM, but also its agglomeration state may be regarded as a key parameter for oral uptake assessment^34,35^. The results in our study showed that the rod-shaped Al_2_O_3_ NMs were present in higher concentrations compared to the spherical Al^0^ NMs in the organs investigated. These findings were in good accordance with the results of uptake investigations of polystyrene NMs on Caco-2 cells^36^. However, this assumption is not always confirmed^37^. Al^0^ NMs might be more easily taken up but also may be excreted faster, whereas the rod shaped structure of Al_2_O_3_ NMs prevents a fast elimination out of the animal. A similar difference between spherical and rod-shaped NMs was also observed for SiO_2_ NM in rats, of which spherical particles were taken up to a higher amount after 2 and 24 h. After 7 days, the rod-shaped particles showed the highest concentrations in the organs^38^. Furthermore, the surface composition might also influence the biokinetic behaviour of the NMs^39^. For Al^0^ NM, a core-shell structure with an Al core and a thin Al oxide layer (2–5 nm) due to passivation with oxygen in the manufacturing process has been confirmed by XRD and element-specific TEM. Al^0^ NMs were shown to contain 85% Al (Table 1). In contrast, Al_2_O_3_ is fully oxidized, with a rather homogenous distribution of Al and oxygen on its surface. Recently we showed that the protein corona formed upon contact with cell culture media is less complex for Al^0^ NM compared to Al_2_O_3_ NM^40^. The different surface properties of NMs facilitate interactions with the surrounding environment, thus the NM, thus influencing bioavailability and tissue retention of the NM^30^. Our results highlight that physico-chemical characteristics including composition, shape, aggregation state and surface composition could affect the kinetics of NMs. As shown in an artificial digestion experiment *in vitro*, AlCl_3_·6H_2_O formed efficiently Al-containing salts and particles in the nano range in the artificial small intestine environment. These newly formed nanoparticles which originated from AlCl_3_·6H_2_O, showed a low solubility comparable to Al^0^ NM^9^. This observation may explain the almost identical uptake behaviour of Al^0^ NM and AlCl_3_·6H_2_O in our study. *In vitro* results from intestinal culture models incubated with the different Al species also showed low uptake rates and no major differences between Al^0^ and Al_2_O_3_ NMs and AlCl_3_·6H_2_O accumulation within the cells^40^. In our study, although the distribution pattern was similar between the two NMs, but the Al concentrations differed significantly. We clearly observed that the Al concentrations found in the organs are not correlated to the total amount of Al given by gavage. In fact, although with the lowest amount of Al in the administered dose, AlCl_3_·6H_2_O was clearly taken up to a higher degree in comparison to NMs (1.9–7.6-fold higher depending on the tissue). The increased uptake of AlCl_3_·6H_2_O might be due to a higher permeability of the intestinal barrier for ions versus particles. However, this was not observed during *in vitro* studies^40^. Nevertheless, compared to the larger agglomerates originating from Al^0^ and Al_2_O_3_ NMs, ions are expected to cross the intestinal barrier more easily. It is worth to mention that the application of Al_2_O_3_ NM with a lower mass fraction of Al than Al^0^ NM, led to a higher accumulation of Al in the organs.

Al^0^ and Al_2_O_3_ NM accumulation between the small and the large intestine differed significantly from each other with a higher concentration found in the colon. The difference of pH between duodenum and colon may impact the agglomeration behaviour of the particles. Moreover, the longer retention time in the colon could also favour their uptake leading to an increased Al amount in the colon^9^.

After passage of the gut –blood barrier, Al was identified in the blood where it possibly is distributed to the systemic organs through the blood flow. Previous work suggested that ionic Al is able to associate with transferrin and other plasma proteins as well as with low molecular weight fractions (citrate, hydroxide)^1^. In our study the blood concentration of AlCl_3_·6H_2_O was relatively low compared to the 1.2-fold higher burden found for the highest dose of Al^0^ NMs. The measured value for Al_2_O_3_ NMs was even 100 times higher than AlCl_3_·6H_2_O (Fig. 4B). Based on the low solubility of Al^0^ and Al_2_O_3_ NMs determined earlier^9^, these results suggests that particulate Al was also transported by blood but probably was better retained in the blood by the formation of a specific protein corona. Especially low molecular weight fractions can circulate between tissue and blood. As AlCl_3_·6H_2_O could potentially interact with low molecular weight proteins, it might reach liver and other organs with a higher efficiency (Fig. 5A, B). In contrast, as shown by protein corona data^40^, Al^0^ NMs and especially Al_2_O_3_ NMs might preferentially interact with higher molecular weight plasma proteins, then limiting their access into tissues and possibly enhancing Al half-life in blood. A few studies investigated the bioavailability and biodistribution of Al-containing NMs after oral treatment in mice. Our results after only three days exposure to Al_2_O_3_ NMs (Fig. 5B) are in good accordance with the biodistribution pattern in liver, kidney and spleen reported after 13 weeks^41^. Another animal study suggested that Al_2_O_3_ NMs was systemically available since liver and kidney impairments were observed after oral uptake^42^.

In conclusion, our *in vivo* study showed a significant uptake of Al from both, Al^0^ and Al_2_O_3_ NMs, following a 3-day oral gavage treatment. Rapid absorption and systemic distribution of Al for all three different forms tested is concluded. Nevertheless, some differences between Al^0^ and Al_2_O_3_ NMs were observed assuming that agglomerate shape and surface composition may play an important role in particle accumulation. In contrast to *in vitro* results from the literature Al_2_O_3_ NM accumulates to a higher extent in comparison to Al^0^ NM in both, intestine and systemic organs. Our findings identify challenges in the extrapolation of NM accumulation, even if the used materials possess a similar chemical composition. An interesting difference to ionic Al was the long retention time of Al levels in blood with Al_2_O_3_ NMs. The low dose study design challenged conventional ICP-MS approaches. In order to increase the sensitivity and robustness of the technique we conducted a matrix calibration in combination with a DRF, which showed superior performance in terms of sensitivity compared to the standard water calibration method. The applied method also eliminated the influence of day to day variation on the calibration. This method enabled the reliable quantification of all Al organ burdens even in the low µg/g range. Our study successfully showed that a respective matrix matched calibration can easily improve the data quality for the evaluation of low dose studies. The approach of statistical evaluation presented here utilizes a DRF to enable robust data analysis in order to obtain resilient data for risk assessment.

## Methods

### Materials

Al^0^ NMs (mean diameter 18 nm (TEM), 99.9%) and Al_2_O_3_ NMs (mean diameter 20 nm (TEM), 99+%) were purchased from Ionic Liquids Technologies GmbH (IoLiTec), Heilbronn, Germany. AlCl_3_·6H_2_O and bovine serum albumin (BSA) were bought from Sigma Aldrich. All other chemicals used in this study were reagent grade.

### Animals and experimental design

All experiments were in accordance with the ethical recommendations of the Directive 2010/63/EU of the European Parliament and were validated by the Anses ethical committee (COMETH). This study was performed to investigate the biodistribution of Al nanomaterials and served as a preliminary dose-finding study for genotoxicity testing, where the doses administered should represent the maximum tolerated doses according to regulatory guidelines. In fact, according to the OECD guidelines 474 and 489 for the micronucleus and comet assays respectively, it is clearly required that the highest dose should be the maximum tolerated dose for a few repeated administrations and that 2 other lower doses must additionally be tested. The experimental design was chosen to fulfil all the above-mentioned criteria.

Male Sprague-Dawley rats (8–10 weeks old, around 200 g) were purchased from Janvier (Saint Berthevin, France). Rats were housed in conventional cages and had free access to water and food. Temperature and humidity were constant with a light/dark cycle of 12 h/12 h. The animals were treated after at least 5 days of acclimatization. Animals (5 per group) were randomly assigned to one of eight groups (including vehicle controls).

Animals were treated by gavage (9.76 ml/kg) at 0 h, 24 h, and 45 h and sacrificed 3 h after the last administration. Al^0^ and Al_2_O_3_ NMs were given at 6, 12.5 and 25 mg/kg body weight (bw), and AlCl_3_·6H_2_O at 25 mg/kg bw in ultra-pure water (UPW) with 0.05% BSA according to the NanoGenoTox protocol and as previously described^30^. UPW with 0.05% BSA was used as negative control.

### Tissue collection and sample preparation

Animals were anesthetized with an intraperitoneal sublethal dose of pentobarbital (60 mg/kg). The following samples were collected: blood, liver, spleen, kidney, duodenum, and colon. The intestinal sections were washed and thoroughly cleaned. A portion of each organ (always a similar part irrespective of treatment) was weighed and rapidly frozen in liquid nitrogen. For the blood samples always 1 ml blood were taken and rapidly frozen in liquid nitrogen. The samples were kept at −80 °C until further processing.

For matrix calibration, a sample of each organ from the controls was spiked with 25 µg/ml of ionic Al. All samples were then digested using a microwave-assisted acid digestion with 69% HNO_3_ and 30% H_2_O_2_ for 30 min at 200°C and 160 bars. The samples were then diluted to a concentration of 500 ng/g. This solution served as stock for the calibration concentrations (1–50 ng/g). All samples were analysed in triplicate.

### Data analysis

The PCA and the one-way ANOVA was carried out in Origin 9.1 (OriginLab Corporation, USA). Microsoft Excel (2016) was used for all other calculations including the F-Test, used for correlation of matrix specific calibrations. The organ weights in combination with the treatment groups were used as input vectors for PCA and ANOVA.

### ICP-MS measurements

Measurements were performed at a quadrupole ICP mass spectrometer (XSeries 2, Thermo Fisher Scientific GmbH, Dreieich, Germany) equipped with a PFA ST Nebulizer, a quartz cyclonic spray chamber and a 2.5 mm quartz injector (all from Thermo Fisher Scientific) using the following isotopes: ^27^Al and, as an internal standard, ^103^Rh. Daily calibrations were performed using ionic standards of Al in a 3.5% HNO_3_ solution ranging from 2 to 500 μg/L. Rh as internal standard was added to each sample. The gas flow for the cool gas and the auxiliary gas were set to 14 L/min, and 0.65 L/min respectively. The sample flow rate was 0.4 mL/min. All isotopes were analysed using the collision cell technique at 5 mL/min collision gas flow (93% He and 7% H_2_).

### Equations

The sample intensities $({\boldsymbol{In}}{{\boldsymbol{t}}}_{{\boldsymbol{Sample}}})$ were corrected by subtracting the number of averaged microwave blanks (≤10) $({\overline{{\boldsymbol{Int}}}}_{{\boldsymbol{Water}}})$:

1 $${\boldsymbol{In}}{{\boldsymbol{t}}}_{{\boldsymbol{corr}}}={\boldsymbol{In}}{{\boldsymbol{t}}}_{{\boldsymbol{Sample}}}-{\overline{{\boldsymbol{Int}}}}_{{\boldsymbol{Water}}}$$

To determine the ${\boldsymbol{DRF}}$, the sensitivity on each day was divided by the sensitivity of the matrix adjusted calibration of that day:

2 $${\boldsymbol{DRF}}=\frac{{\boldsymbol{sensitivit}}{{\boldsymbol{y}}}_{{\boldsymbol{sample}}{\boldsymbol{day}}}}{{\boldsymbol{sensitivit}}{{\boldsymbol{y}}}_{{\boldsymbol{matrix}}{\boldsymbol{calibration}}{\boldsymbol{day}}}}$$

DRF was then used to correct the intensity values:

3 $${\boldsymbol{In}}{{\boldsymbol{t}}}_{{\boldsymbol{sample}},{\boldsymbol{corr}}}={\boldsymbol{In}}{{\boldsymbol{t}}}_{{\boldsymbol{corr}}}/{\boldsymbol{DRF}}$$

Dividing Eq. 1 by Eq. 2 considering the Al sensitivity in the corresponding matrix, the used organ mass $({{\boldsymbol{m}}}_{{\boldsymbol{org}}})$ and the recovery after the microwave digestion $({{\boldsymbol{R}}}_{{\boldsymbol{MW}}})$ results in the Al organ burden per gram organ:

4 $${\boldsymbol{c}}({\boldsymbol{Al}})=[([{\boldsymbol{In}}{{\boldsymbol{t}}}_{{\boldsymbol{sample}},{\boldsymbol{corr}}}/{{\boldsymbol{S}}}_{{\boldsymbol{matrix}}}])/{{\boldsymbol{m}}}_{{\boldsymbol{org}}}]/{{\boldsymbol{R}}}_{{\boldsymbol{MW}}}$$

LOD and LOQ were determined using the blank value method^43^. The data of the control group (5 animals) were used for the determination of the lowest detectable signal. The intensities obtained were converted into concentrations using the matrix calibrations (Table 2). The data for the different groups did not follow a normal distribution. Therefore, median and interquartile ranges (IQR) of the control group for each organ were used to calculate the LOD and LOQ. A 1.5 × IQR corresponds to a standard deviation of 3σ:

5 $${\boldsymbol{LOD}}={\boldsymbol{Median}}+{\bf{1.5}}\,\ast \,{\boldsymbol{IQR}}$$

6 $${\boldsymbol{LOQ}}={\boldsymbol{Median}}+{\bf{5}}\,\ast \,{\boldsymbol{IQR}}$$

Al concentrations $({{\boldsymbol{c}}}_{{\boldsymbol{Al}}{\boldsymbol{NM}}})$ were normalized on the AlCl_3_·6H_2_O concentration $({{\boldsymbol{c}}}_{{\boldsymbol{AlC}}{{\boldsymbol{l}}}_{3}})$ (Figs. 3 and 4), corrected by the mass fraction of AlCl_3_·6H_2_O per rat $({{\boldsymbol{m}}}_{{\boldsymbol{AlC}}{{\boldsymbol{l}}}_{3}})$ and the corresponding NM species $({{\boldsymbol{m}}}_{{\boldsymbol{Al}}{\boldsymbol{NM}}})$:

7 $${\boldsymbol{median}}\,{\boldsymbol{normalized}}\,{\boldsymbol{Al}}\,{\boldsymbol{concentration}}=\frac{{{\boldsymbol{c}}}_{{\boldsymbol{Al}}{\boldsymbol{NM}}}\,\ast \,{{\boldsymbol{m}}}_{{\boldsymbol{AlC}}{{\boldsymbol{l}}}_{3}}\,}{{{\boldsymbol{c}}}_{{\boldsymbol{AlC}}{{\boldsymbol{l}}}_{3}}\,\ast \,{{\boldsymbol{m}}}_{{\boldsymbol{Al}}{\boldsymbol{NM}}}}$$

Therefore, the term “Al concentration” refers to the concentration of Al measured by ICP-MS. The median normalized Al concentration is only used for subsequent evaluation of Al organ burdens.

## Supplementary information

Supplementary information.

## Data Availability

The ICP-MS data sets can be obtained by the authors upon individual request.
