# Supplementary material for: Aluminum and aluminum oxide nanomaterials uptake after oral exposure - a comparative study
Source: Sci Rep. 2020 Feb 14;10:2698. doi: 10.1038/s41598-020-59710-z (PMC7021764; doi:10.1038/s41598-020-59710-z)
Supplement: Supplementary file 1 — Supplementary information. [file 41598_2020_59710_MOESM1_ESM.docx]

**Aluminum and aluminum oxide nanomaterials uptake after oral exposure - a comparative study**

Benjamin C. Krause (Benjamin-christoph.krause@bfr.bund.de)*^#1^

Fabian L. Kriegel (Fabian.kriegel@bfr.bund.de)^#1^

Daniel Rosenkranz (Daniel.rosenkranz@bfr.bund.de) ^#1^

Nadine Dreiack (Nadine.dreiack@bfr.bund.de)^1^

Jutta Tentschert (Jutta.tentschert@bfr.bund.de)^1^

Harald Jungnickel (Harald.jungnickel@bfr.bund.de)^1^

Pegah Jalili (jalilipegah@gmail.com)^2^

Valerie Fessard (Valerie.fessard@anses.fr)^2^

Peter Laux (Peter.laux@bfr.bund)^1^

Andreas Luch (Andreas.luch@bfr.bund.de)^1^

^1^German Federal Institute for Risk Assessment (BfR), Department of Chemical and Product Safety, Max-Dohrn-Straße 8-10, 10589 Berlin, Germany.

^2^ANSES, French Agency for Food, Environmental and Occupational Health and Safety, Fougères Laboratory, 10B rue Claude Bourgelat, 35306, Fougères Cedex, France.

^#^= Authors contributed equally

^*^= Corresponding author

**Supplement**

Supplementary Table S1: One-way ANOVA for organ weights based on the different treatment groups.

| **Group** | **N Analysis** | **Mean [mg]** | **Standard deviation[mg]** | **SE of Mean [mg]** |  |
| --- | --- | --- | --- | --- | --- |
| 1 | 5 | 1512 | 570 | 255 |  |
| 2 | 5 | 1634 | 375 | 168 |  |
| 3 | 5 | 1614 | 329 | 147 |  |
| 4 | 5 | 1533 | 391 | 175 |  |
| 5 | 5 | 1442 | 252 | 112 |  |
| 6 | 4 | 1358 | 534 | 267 |  |
| 7 | 5 | 1462 | 404 | 181 |  |
| 8 | 5 | 1526 | 396 | 177 |  |
|  | **DF** | **Sum of Square [mg²]** | **Mean Square [mg]** | **F Value** | **Prob>F** |
| **Model** | 7 | 261943 | 37420 | 0.219 | 0.978 |
| **Error** | 31 | 5292160 | 170715 |  |  |
| **Total** | 38 | 5554110 |  |  |  |

1: control; 2: Al 6.25 mg/kg bw; 3: Al 12.5 mg/kg bw; 4: Al 25 mg/kg bw; 5: Al_2_O_3_ 6.25 mg/kg bw; 6: Al_2_O_3_ 12.5 mg/kg bw; 7: Al_2_O_3_ 25 mg/kg bw; 8: AlCl_3_·6H_2_O 25 mg/kg bw; no significant difference for p ≤ 0.05.





Supplementary Fig. S1: Principal Component Analysis for organ weights based on different treatment groups.





Supplementary Fig. S2: Calibration curves for matrices investigated in this study.

Supplementary Table S2: Data normalization for Al concentration. The example was calculated for liver according to the equation 7.

|  | dose per day  [mg/kg bw] | total given  Al amount  per rat [mg] | Al concentrations per gram liver [µg/g] | normalized to total given Al amount [‰] | normalized to 1.7 mg/kg AlCl_3_·6H_2_O [%] |
| --- | --- | --- | --- | --- | --- |
| Al^0^ NM | 6.25 | 3.19 | 0.52 | 0.16 | 32.65 |
|  | 12.5 | 6.38 | 0.26 | 0.04 | 8.16 |
|  | 25 | 12.75 | 0.49 | 0.04 | 8.16 |
| Al_2_O_3_ NM | 6.25 | 1.99 | 1.48 | 0.74 | 151.02 |
|  | 12.5 | 3.97 | 1.17 | 0.29 | 59.18 |
|  | 25 | 7.94 | 1.07 | 0.13 | 26.53 |
| AlCl_3_·6H_2_O | 25 | 1.68 | 0.83 | 0.49 | 100 |

Supplementary Table S3: Data normalization for Al concentration. The example was calculated for the duodenum according to the equation 7.

|  | dose per day  [mg/kg bw] | total given  Al amount  per rat [mg] | Al concentrations per gram duodenum [µg/g] | normalized to total given Al amount [‰] | normalized to 1.7 mg/kg AlCl_3_·6H_2_O [%] |
| --- | --- | --- | --- | --- | --- |
| Al^0^ NM | 6.25 | 3.19 | 2.21 | 0.69 | 85.19 |
|  | 12.5 | 6.38 | 0.86 | 0.13 | 16.05 |
|  | 25 | 12.75 | 1.13 | 0.09 | 11.11 |
| Al_2_O_3_-NM | 6.25 | 1.99 | 1.82 | 0.91 | 112.35 |
|  | 12.5 | 3.97 | 1.27 | 0.32 | 39.51 |
|  | 25 | 7.94 | 1.29 | 0.16 | 19.75 |
| AlCl_3_·6H_2_O | 25 | 1.68 | 1.36 | 0.81 | 100 |





Supplementary Fig. S3: Boxplots showing the concentration of Al per organ. Data are given for all treatment groups with median and 1.5 IQR, including outliers. A: Al concentration in duodenum; B: Al concentration in colon; C: Al concentration in liver; D: Al concentration in kidney; E: Al concentration in spleen; F: Al concentration in blood.
